# Combining in silico prediction and ribosome profiling in a genome-wide search for novel putatively coding sORFs

**DOI:** 10.1186/1471-2164-14-648

**Published:** 2013-09-23

**Authors:** Jeroen Crappé, Wim Van Criekinge, Geert Trooskens, Eisuke Hayakawa, Walter Luyten, Geert Baggerman, Gerben Menschaert

**Affiliations:** 1 Lab of Bioinformatics and Computational Genomics (BioBix), Department of Mathematical Modelling, Statistics and Bioinformatics, Faculty of Bioscience Engineering, Ghent University, 9000 Ghent, Belgium; 2 Research Group of Functional Genomics and Proteomics, KU Leuven, 3000 Leuven, Belgium; 3 Department of Pharmaceutical and Pharmacological Sciences, Faculty of Medicine, KU Leuven, 3000 Leuven, Belgium; 4 VITO Nv, 2400 Mol, Belgium – CFP, Center For Proteomics, 2020 Antwerpen, Belgium

**Keywords:** Micropeptide, Small open reading frame, *Mus musculus*, Genome-wide, Ribosome profiling, LincRNA, sORF, ncRNA, Bioactive peptide

## Abstract

**Background:**

It was long assumed that proteins are at least 100 amino acids (AAs) long. Moreover, the detection of short translation products (e.g. coded from small Open Reading Frames, sORFs) is very difficult as the short length makes it hard to distinguish true coding ORFs from ORFs occurring by chance. Nevertheless, over the past few years many such non-canonical genes (with ORFs < 100 AAs) have been discovered in different organisms like *Arabidopsis thaliana*, *Saccharomyces cerevisiae*, and *Drosophila melanogaster*. Thanks to advances in sequencing, bioinformatics and computing power, it is now possible to scan the genome in unprecedented scrutiny, for example in a search of this type of small ORFs.

**Results:**

Using bioinformatics methods, we performed a systematic search for putatively functional sORFs in the *Mus musculus* genome. A genome-wide scan detected all sORFs which were subsequently analyzed for their coding potential, based on evolutionary conservation at the AA level, and ranked using a Support Vector Machine (SVM) learning model. The ranked sORFs are finally overlapped with ribosome profiling data, hinting to sORF translation. All candidates are visually inspected using an in-house developed genome browser. In this way dozens of highly conserved sORFs, targeted by ribosomes were identified in the mouse genome, putatively encoding micropeptides.

**Conclusion:**

Our combined genome-wide approach leads to the prediction of a comprehensive but manageable set of putatively coding sORFs, a very important first step towards the identification of a new class of bioactive peptides, called micropeptides.

## Background

Classical bioactive peptides are cleaved from larger precursor proteins that have a signal sequence at their N-terminus [1,2]. As a consequence they are targeted into the secretory pathway and once extra-cellular play a –autocrine, paracrine, or endocrine- signaling role for example by activating G-protein coupled receptors of neighboring or more distant cells. More recently new concepts and different classes of bioactive peptides are described. A first class consists of intracellular peptides having a regulatory effect on cell signaling [3]. Another recent class (non-classical) consists of peptides that are not processed in the secretory machinery. One type of this latter class is immediately translated from small open reading frames (sORFs; < 100 AA) [2,4,5]. Since they lack an N-terminal signal sequence they are in principle set free in the cytoplasm immediately after translation. From hereon, these are denoted as micropeptides.

Although some members of this new category could already be linked to important embryonic and morphogenetic functions in plants as well as in animals [2], micropeptide research is not yet widespread. Hundreds of novel sORFs, supported by transcriptional evidence, could be identified in the *Saccharomyces cerevisiae* genome, most of them with sequence similarity to ORFs in other organisms. Comprehensive analysis of one specific sORF, termed *smORF2* even shows sequence conservation between yeast and human [6,7]. In *Arabidopsis thaliana* the *POLARIS (PLS)* polypeptide gene was identified in a promoter trap transgenic line predominantly showing expression in the embryonic basal region and affecting root growth and leaf vascularization [8]. Next to this already characterized *PLS* peptide, hundreds of other novel possible coding sORFs were identified in intergenic regions of the *Arabidopsis thaliana* genome [9]. Other plant micropeptides have been examined: the recessive mutation of *Brick1* in maize leads to several morphological defects of leaf epithelia, and *Enod40* is a polycistronic micropeptide translated in soybean playing a distinct role in the control of sucrose use in nodules [2,10,11].

In animals, a handful of functional micropeptides have also been discovered. An evolutionary conserved micropeptide was identified in *Drosophila* and referred to as *polished rice (pri)* or *tarsal-less (tal)*, while the *Tribolium* orthologue is called *mille-pattes (mlpt)*[4,12,13]. These *tal* (or *pri*) peptides (11 AAs long) control epidermal differentiation by modifying the transcription factor *Shavenbaby (Svb)*[5]. Increasing evidence suggests that these so-called micropeptides are also present in higher animals, including mammals. Analyzing the mouse genome using CRITICA [14] confirmed the existence of many short ORFs, roughly accounting for 10% of the mouse proteome [15]. Also, a recent paper by Ingolia *et al.* defines a new class of short polycistronic ribosome-associated coding RNAs (sprcRNAs) encoding small proteins [16]. In human cells, Slavoff *et al.* identified 90 sORF-encoded polypeptides (SEP) of which 86 were previously uncharacterized [17].

The past decade has seen considerable advances in both sequencing technology and computing infrastructure, resulting in ever-more annotated genomes already from over a hundred eukaryotic species [18]. Such efforts are valuable to the discovery of sORFs putatively encoding micropeptides for example by providing us with a high-resolution view of the developmental transcriptome, identifying thousands of newly transcribed regions (NTRs) [19,20] or a conserved set of long intervening non-coding RNAs (linRNAs) and other non-coding RNAs (ncRNAs) [21,22] in different species. Furthermore, new sequencing methodologies emerge. Ribosome profiling, a recently described technique, based on deep sequencing of ribosome-protected mRNA fragments, enables the high-precision and genome-wide monitoring of translation [16,23,24]. Such ribosome profiling experiments performed on mouse embryonic stem cells (mESCs) [16] and human embryonic kidney 293 (HEK293s) cells [24], further strengthen the theory that short un-annotated RNA sequences or ribosome footprints can encode micropeptides, especially because the length of ORFs in the NTRs is very frequently below 100 AAs in these studies. Of particular interest in recent literature are sORFs within lincRNAs as research points to the existence of such RNAs expressing different short polypeptides [25]. However the debate on the extent of their peptide coding capacities is ongoing [26,27].

Although huge numbers of novel transcripts are documented in every transcriptome sequencing project, gene-prediction is still a challenge, especially when looking for functional sORFs [28]. Until recently, most gene-prediction tools arbitrarily applied a minimum sequence length cutoff (e.g. 100 AAs), reducing the likelihood of false positive predictions [29]. False negative ratios also increase when trying to discover small coding sequences as they lack splicing signals on either side of the single exon and show a decreasing signal-to-noise ratio as the size of the coding region decreases [30,31]. In an attempt to circumvent these limitations sORFfinder, a software package to identify specifically sORFs with high coding potential [32] was devised. sORFfinder makes use of the nucleotide composition bias between coding and non-coding sequences to evaluate the coding potential of those functional sORFs [9]. However, genome-wide searches for sORFs in higher eukaryotes are still seen as a computational burden: thus no such data exist for any higher eukaryote [24,33].

To the best of our knowledge, a systematic genome-wide study scanning for sORFs that encode small peptides has not yet been performed for a mammalian. A first genome-wide search for sORFs has been undertaken for *Saccharomyces cerevisiae*. A combination of *in silico* and experimental approaches proves the existence of at least 299 sORFs in the yeast genome, accounting for up to 5% of the protein-coding genes [7]. In *Arabidopsis thaliana*, a systematic search for sORFs revealed the potential existence of 3,241 coding sequences for which evidence for transcription or purifying selection is available [9]. A recent study describes a systematic search for putatively functional sORFs in euchromatic regions of *Drosophila melanogaster*, postulating the existence of at least 401 sORFs coding for small peptides [33]. In this report we combine a genome-wide *in silico* search strategy and the specific characteristics of ribosome profiling data in a search for sORFs putatively encoding functional micropeptides in the model organism *Mus musculus.*

## Results

### Genome-wide identification of sORFs

The genome-wide search for sORFs with sORFfinder resulted in the prediction of 2,414,358 single-exon sORFs with high coding potential, out of a total pool of 40,704,347 sORFs (see Table 1). The strand-specific genomic location, sequence, and coding potential score (calculated by sORFfinder) were, like all subsequently obtained data, stored in a MySQL relational database (see Methods).

**Table 1:** Basic sORF characteristics

**Chromosome**	**Length (bp)**	**Total number of sORFs**	**One sORF per number of bps (bp)**	**Number of sORFs with high coding potential (sORFfinder)**	**One high coding sORF per number of bps (bp)**
1	197,195,432	3,070,032	128	160,770	2,453
2	181,748,087	2,830,394	128	176,654	2,058
3	159,599,783	2,507,691	127	124,217	2,570
4	155,630,120	2,385,489	130	155,419	2,003
5	152,537,259	2,335,678	131	158,789	1,921
6	149,517,037	2,342,614	128	130,505	2,291
7	152,524,553	2,235,697	136	146,314	2,085
8	131,738,871	1,990,727	132	134,093	1,965
9	124,076,172	1,910,809	130	126,743	1,958
10	129,993,255	2,024,292	128	121,848	2,134
11	121,843,856	1,845,184	132	142,610	1,709
12	121,257,530	1,875,766	129	109,403	2,217
13	120,284,312	1,867,333	129	108,919	2,209
14	125,194,864	1,959,570	128	102,912	2,433
15	103,494,974	1,599,415	129	101,315	2,043
16	98,319,150	1,528,958	129	81,787	2,404
17	95,272,651	1,441,669	132	102,617	1,857
18	90,772,031	1,404,482	129	80,524	2,255
19	61,342,430	912,412	134	65,280	1,879
X	166,650,296	2,594,439	128	82,073	4,061
Y	15,902,555	41,696	762	1,566	20,310
**Total**	**2,654,895,218**	**40,704,347**	**130**	**2,414,358**	**2,199**

Overview of putatively coding sORFs grouped by Mus *musculus* chromosomes*,* showing the total number and the distribution of sORFs for each chromosome, as well as the number and distribution of sORFs with high coding potential according to sORFfinder.

**Figure 1:**
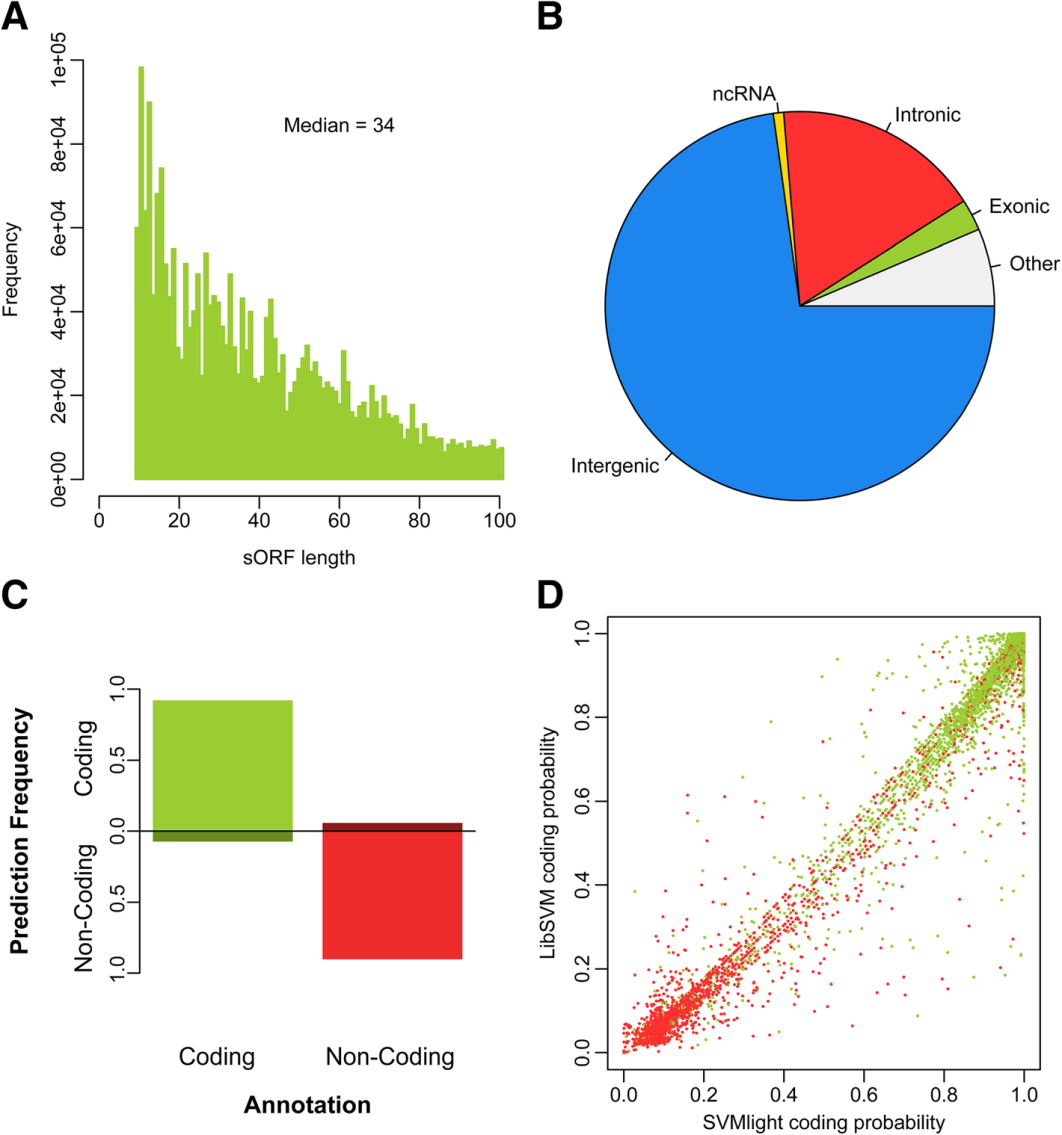
**Overview of the *in silico *coding sORF prediction. (A)** Histogram of the total number of sORFs depicted by ORF length (in AA). **(B)** Distribution of sORFs according to their genomic location. sORfs overlapping more than one different category are grouped as “others”. **(C)** Evaluation of the sORF coding probability. The fractions of annotated and predicted coding and non-coding sORFs within the test dataset are plotted. **(D)** Visual representation of the classification of all 9,612 test subjects, based upon both SVMs (SVMlight and libSVM). True coding sORFs are depicted in green and true non-coding in red (see Additional file 1: Figure S2).

The number of sORFs with a specific length tends to increase with decreasing length (Figure 1A). This comes as no surprise since short nucleotide sequences with ORF-like qualities may easily appear by chance. Including sORFs with a length smaller than 10 AAs would thus exponentially increase the pool of sORFs under investigation and make further computational analysis much more demanding or even impossible. If we look at the total number of sORFs found by sORFfinder, and assume a random and even distribution across the genome, we see that sORFs are distributed evenly in the different autosomes with circa 1 sORF every 130 bp (Table 1). There is slightly more variation in distance between adjacent sORFs (again assuming even distribution) when looking only at those with high coding potential, with on average 1 sORF every 2,200 bp. As can be seen in Table 1, the sex chromosomes tend to deviate from these averages with sparser (high coding) sORF appearances.

All resulting sORFs from sORFfinder were overlapped with the genome-wide Ensembl annotation (NCBIM37, annotation version 66). As can be seen in Figure 1B and associated Table 2, most of the sORFs are located in intergenic regions as these take up most of the genome. Although many sORFs are located in intronic regions, this is less then would be expected (assuming an even distribution of sORFs throughout the genome) *(Exact binomial test p < 2.2e-16).* In the same way, almost double the number of sORFs were found in exonic regions as would be expected under an even distribution *(Exact binomial test p < 2.2e-16)*[34]. These observations indicate that sORFfinder succeeds in making a good distinction between coding and non-coding sORFs.

**Table 2:** Coding potential of sORFs in different genomic locations

**Genomic location**	**# of sORFs**^**a**^	**Coding sORFs**^**b**^	**Pcod > 0.9**^**c**^	**Pcod > 0.99**^**c**^	**Ribo sORFs**^**d**^	**Coding Ribo sORFs**^**e**^
ncRNA	20,810	9,922	6,443	1,100	528	401
Exonic	63,180	34,063	21,546	10,872		
Other	155,633	80,891	37,730	9,894		
Intronic	417,277	34,845	14,582	2,361		
Intergenic	1,757,458	223,235	107,567	27,371	226	89

Number of sORFs divided per genomic region and for which certain *in silico* and/or expression evidence can be found. Included are total number of sOFs with high coding potential (according to sORFfinder), number of sORFs having scores above certain thresholds (according to SVM analysis), number of sORFs which show ribosome profiling expression and number of sORFs for which *in silico* coding as well as expression evidence is available.

^a^ Total number of sORFs with high coding potential according to sORFfinder.

^b^ Total number of sORFs classified as coding by SVM^light^.

^c^ Pcod is the coding probability score as predicted by SVM^light^.

^d^ sORFs with mapped ribosome profiles, attaining sequence read coverage > 75% of the total ORF (based on cycloheximide treatment), and ribosome profile hits at the ORF start site (based on harringtonine treated samples).

^e^ Ribo sORFs (see under ^d^ for description) classified as coding by SVM^light^.

### Peptide conservation based on UCSC multiple species alignment

To assess their peptide-coding potential, all sORFs were analyzed using a multi-species alignment of 8 vertebrate species (See Methods). For each sORF a number of basic peptide conservation characteristics were deduced and gathered (see Additional file 1: Figure S1 for an example). Each overview contains the DNA and AA sORF alignment for all conserved species. Each pair-wise alignment for all conserved species is further analyzed to obtain the specific coding potential characteristics. In this way, we obtained the number of species in which the ORF length as well as the start and stop codons are conserved. Furthermore all mutations between the alignments are analyzed for their synonymous or non-synonymous nature, permitting the calculation of synonymous and non-synonymous substitution rates (Ks and Ka, respectively). In a last step, K_a_/K_s_ values for each pair-wise alignment were calculated. All obtained characteristics and positional info regarding all the sORFs are stored in a data matrix and are available via Additional file 1.

### Classification and ranking

We used an SVM approach to classify the sORFs into a coding and non-coding group based on all aforementioned characteristics. After training the SVM on 4/5^th^ of the data and testing the SVM on the remainder, we reached a correct classification for up to 93% of the test subjects, with a false positive rate not exceeding 4% (Figure 1C). Classification via SVM^light^ was also verified by running the same analysis using a second SVM package (libSVM). The outcome of both SVM packages shows a very good correlation (see Figure 1D and 1E), proving the robustness of the SVM approach.

### Cross-validation with mESC ribosome profiling data

**Figure 2:**
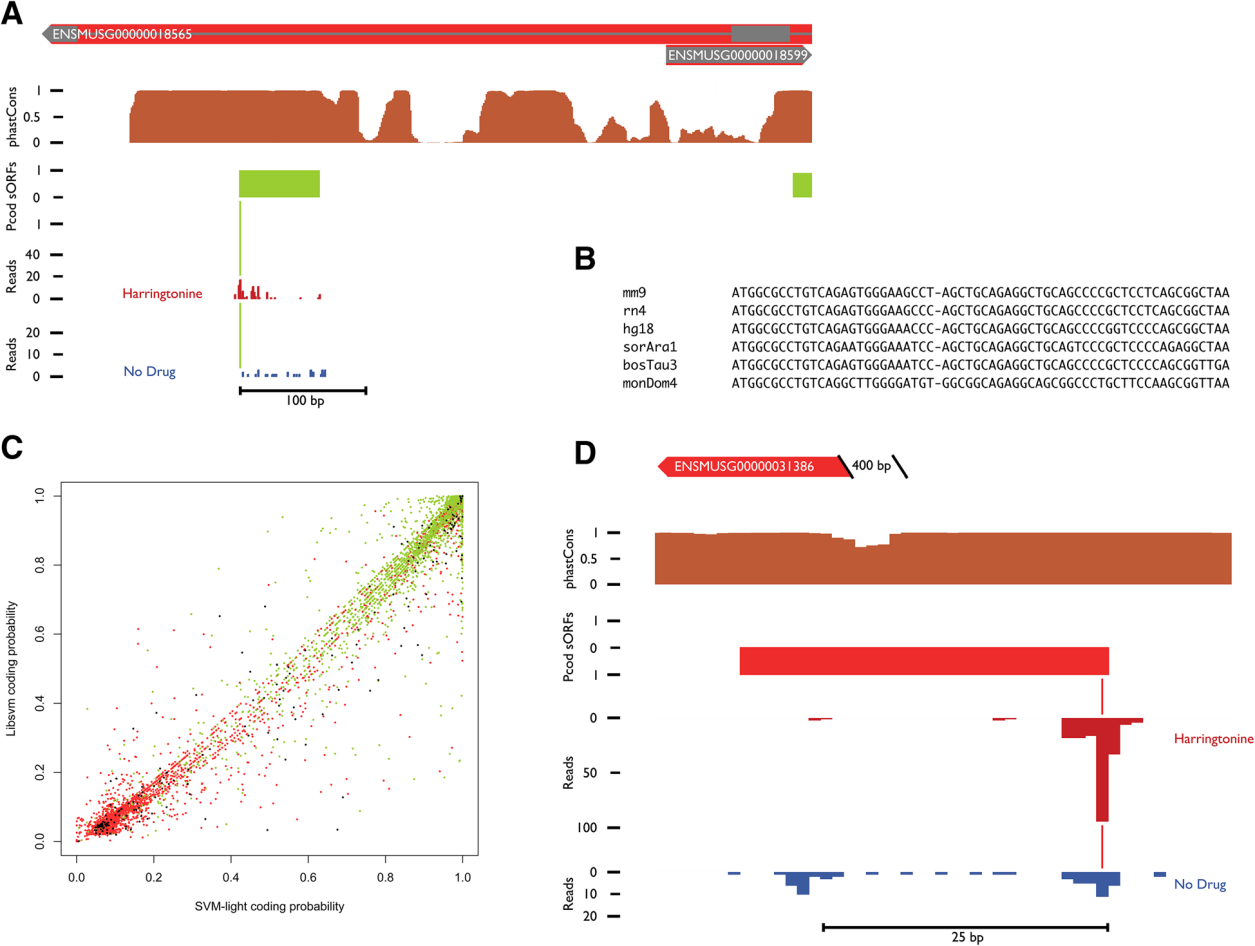
**The combined approach identifies many putatively functional sORFs in intergenic regions. (A)** Visual representation of the intergenic sORF located on the forward strand of chromosome 11 (69,794,326-69,794,388) based on data from the H2G2 genome browser. **(B)** DNA multiple alignments for the intergenic sORF presented in Figure 2A and based on the 8 species under investigation from the UCSC mm9 multi-species alignment. **(C)** Visual representation of the overlap between intergenic sORFs with ribosomal profiling evidence and the classified test subjects. True coding sORFs are depicted in green and true non-coding in red (see Additional file 1: Figure S2), black dots represent the intergenic sORFs. Classification and presentation are based on the coding probability scores from the 2 SVMs used during the analysis (See Methods). **(D)** Visual representation of the intergenic sORF located on the reverse strand of chromosome X (71,212,050-71,212,082) based on data from the H^2^G^2^ genome browser. The sORF is located approximately 400 bp upstream of a known protein-coding gene (*Hcfc1*).

**Figure 3:**
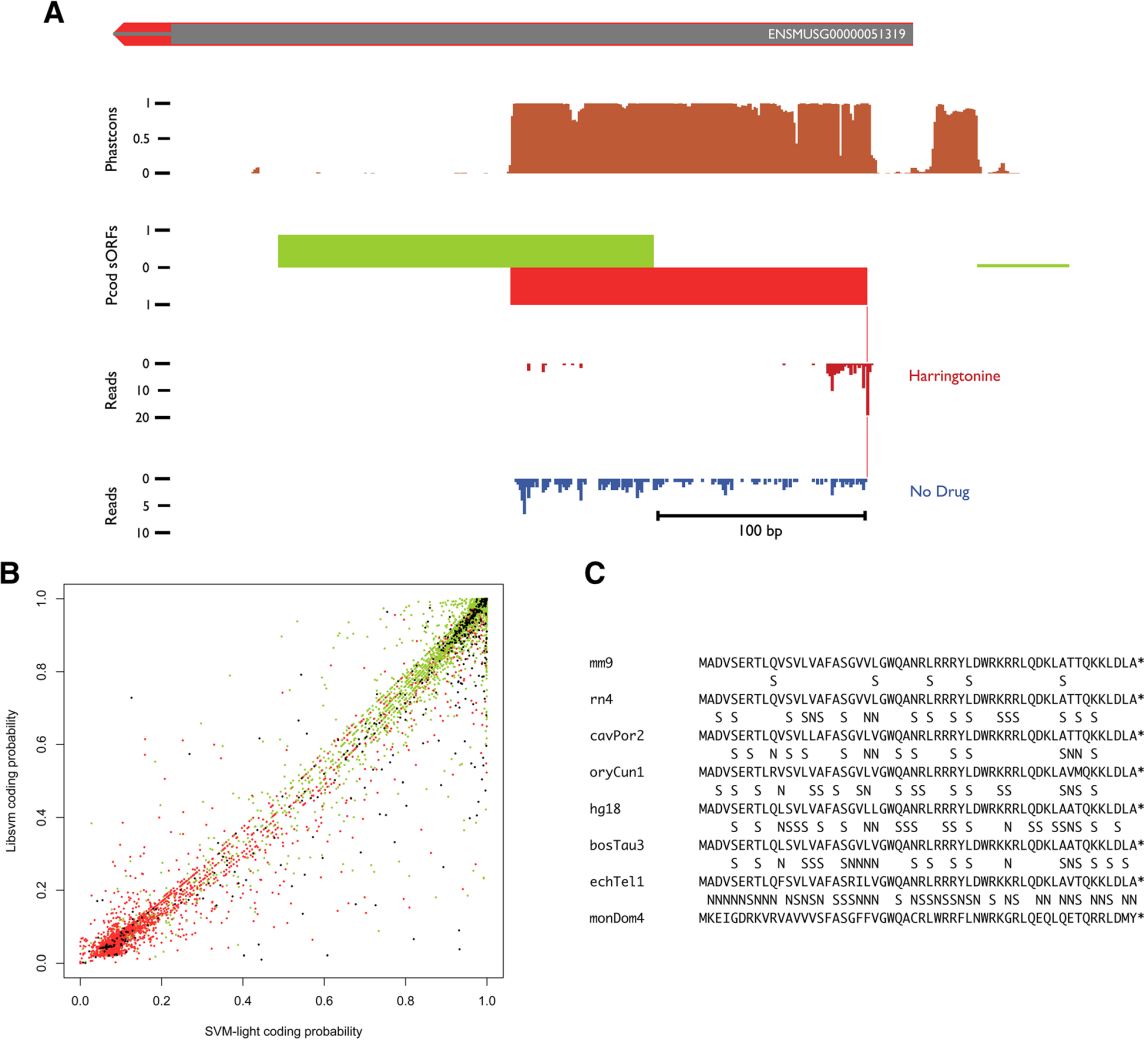
**The combined approach identifies many putatively functional sORFs in ncRNA regions. (A)** Visual representation of the lincRNA overlapping sORF located on the reverse strand of chromosome 2 (127,618,033 – 127,618,203) based on data from the H2G2 genome browser. **(B)** Visual representation of the overlap between ncRNA overlapping sORFs with ribosomal profiling evidence and the classified test subjects. True coding sORFs are depicted in green and true non-coding in red (see Additional file 1: Figure S2), black dots represent the ncRNA overlapping sORFs. Classification and presentation are based on the coding probability scores from the 2 SVMs used during the analysis (See Methods). **(C)** AA multiple alignments for the lincRNA overlapping sORF presented in Figure 3A and based on the 8 species under investigation from the UCSC mm9 multi-species alignment. Next to the AA sequences for each species, a synonymous (S) versus non-synonymous (N) annotated conservation line is added for better interpretation (see Additional file 1: Figure S1 for the complete sORF overview file).

Even with very stringent parameters this genome-wide *in silico* prediction approach gives rise to hundreds, even thousands of possibly interesting sequences (Table 2). We reasoned that a combined approach incorporating also biologically relevant data next to the *in silico* analysis should lead to a more meaningful set of sORFs, at the same time overcoming several approach-specific limitations (see Discussion). Therefore we reanalyzed ribosome profiling data obtained from a mouse Embryonic Stem Cells (mESC) sample [16]. The sequencing reads were uniquely mapped to sORFs located in intergenic or ncRNA regions. Retaining only those sORFs that overlap with ribosome profiles at their start position in the harringtonine treated sample data and that have a sequence read coverage of at least 75% relative to the untreated sample data, led to a set of 226 intergenic sORFs and 528 sORFs located in ncRNA regions. Looking only at lincRNA sORFs, as data points to their expression in these regions [16], further decreases the sample size to 35 sORFs. An overlap of the aforementioned intergenic and ncRNA sORFs with the SVM training data can be seen in Figures 2C and 3B, respectively. In combination with the conservation characteristics from the *in silico* prediction, this gives rise to a set of sORFs that (A) show a high coding probability score based on the aforementioned SVM approach and (B) overlap with biologically relevant ribosomal profiles (see Table 2). The expression of the ncRNA and intergenic sORFs with coverage > 75% and harringtonine treated ribosome profile occupancy was also compared with the CHX treated embryoid body sample data [16] (see Additional file 2: Figure S3). Further research on a case-by-case basis will be necessary to evaluate and interpret the differential expression between different developmental stages.

### Visualization

All sORFs are mapped on the reference genome and made accessible through an in-house developed H2G2 genome browser, (see Additional file 1 for login credentials). Next to the sORF information, static visualization tracks are added depicting genomic annotation from Ensembl, phastCons conservation scores and other relevant information. Experimental ribosomal profiling data are incorporated using individual tracks for every analysis on the different samples (with or without harringtonine treatment). Together with the UCSC multi-species alignment such visual representation of all available information makes interpretation of the results far easier. For example, Figures 2 and 3 depict some newly discovered sORFs (from the intergenic and ncRNA pool, respectively) within the H2G2 genome browser.

## Discussion

It is generally accepted that looking for conservation across species is one of the best strategies for finding functional sequences. In this study, the phastCons conservation score in combination with specific peptide conservation characteristics derived from the UCSC multi-species alignment [35] is used to predict the coding probability of sORFs. We reasoned that the *in silico* method in combination with experimental translational evidence would lead to an even more robust, stringent, and more importantly, *in vivo* validated prediction. We therefore combined the *in silico* approach with information from a reanalyzed ribosome profiling study on mESCs. Ribosome profiling is an experimental method to monitor *in vivo* translation by deep sequencing of ribosome-protected mRNA fragments [16] thus reflecting ribosomal occupancy.

In this report, we show that the combination of both the *in silico* prediction and the *in vivo* data leads to the discovery of many new putatively coding sORFs in the mouse genome (see Additional file 2). The identified sORFs have a high AA conservation in multiple species, show ORF translation (based on cycloheximide-treated mESC line ribosome profiling experiments) and moreover exhibit a ribosome profile peak at their start codon (based on harringtonine-treated mESC line experiments). When analyzing the overlapping data, we specifically investigated sORFs within ncRNA and intergenic regions. Although we are convinced that sORFs overlapping other regions constitute interesting study objects, we concentrated on the aforementioned regions in this study for the following reasons.

The first eukaryotic micropeptide, *tarsal-less* or *pri*, was discovered in a ncRNA-annotated region [5]. Since ORFs shorter than 100 AAs have long been disregarded in the past, it is possible that other ncRNAs are in fact coding for small peptides, making this set of sORFs especially interesting [36]. More recent studies also point to the expression of specific small peptides encoded within lincRNAs in mammals [16,24,25,27]. The described results (see Table 2 and Additional file 2) strengthen the idea that some ncRNA regions actually contain putatively coding sORFs. Investigating the sORFs within annotated lincRNA regions still yields very well-conserved and expressed sORFs (see Figure 3A and 3C). Further analysis of the conservation of the sORF presented in Figure 3, overlapping a lincRNA (*1500011K16Rik*) was done by means of a BLAST search against the human genome. This resulted in the identification of 1 region within the second and biggest exon of lincRNA *LINC00116*, part of the GENCODE annotation [37], further confirming the significance of our findings (see Additional file 1: Figure S4). The fact that lincRNA expression in mESCs tend to be low [16], lincRNA are rarely translated in different cell lines [27] and that known micropeptides have a very narrow expression window in time as well as in space [5], suggests that many putatively coding sORFs remain to be detected.

Next to the aforementioned ncRNA sORFs, the set of sORFs located in intergenic regions was also investigated, revealing dozens of highly conserved sORFs with ribosome profiling experimental validation. One of the more striking things we see in our results on intergenic sORFs is that a lot of the high-scoring and expressed intergenic sORFs are located close to known protein-coding genes (see Figure 2A and 2D). Most of these can be found between 1 kb and 100 bp upstream of the 5′ untranslated region (UTR). Several explanations can be formulated for this phenomenon, one of the more obvious ones of course being misannotation of the known gene close to the sORF. First, one could argue the existence of additional exons, upstream of the already annotated ones, which have been overlooked so far. This could give an explanation for some of the identified sORFs, especially those showing low conservation at, and around, the stop codon (translation would not reach this stop triplet since a prior splicing event would prevent this). Secondly, sORFs are sometimes located within 1–200 bp from each other, mostly only measuring 10 – 20 AAs in length, but with high stop codon conservation. The likeliness of multiple splice sites within these very short, and highly conserved, sORFs seems at least debatable. Misannotation is not unique to aforementioned exonic regions, as it could also have happened at the level of the 5′ UTR, giving rise to sORFs possible being uORFs (described as regulators of the translation of the downstream coding sequence [38]). However, keeping in mind all characteristics of the identified sORFs, it cannot be ruled out that some sORFs in the proximity of known genes could give rise to a new class of functional peptides playing a (regulatory) role that still has to be explored. In plants, increasing data indicate that sORFs play diverse roles in regulating expression and in this way participate in various cellular processes [39]. Also, research from [40] points to the existence of thousands of previously unknown bovine ncRNAs in the proximity of known genes, possibly encoding sORFs. Their set-up specifically removed ncRNA sequences with ORFs longer than 50 AAs; so small putatively translated ORFs could still be present. They also performed a correlation analysis on expression levels between these intergenic ncRNAs and protein coding genes, revealing significant correlation for many transcripts, supporting the hypothesis that these ncRNA sORFs could have a regulatory function.

The *in silico* generated prediction score, as outlined in the results and methods sections, also has its limitations. Using sORFfinder as a first filtering step introduces false positive as well as false negative sORFs [32]. Although we are working on ways to eliminate the use of this tool, for the moment, this initial step is still indispensable (because of a too high computational workload) to get the total number of sORFs down in a sensible way, based on the coding index. The prediction furthermore greatly depends on the correctness of the multiple species sequence alignments. In cases where a sORF has no, or very few aligned sequences (within the set of 8 pair-wise alignments taken into account in this study), the SVM assesses the coding probability mainly on the phastCons conservation score. This score, reflecting DNA conservation, already has a considerable impact on the overall prediction as can be seen in Additional file 1: Figure S5. Distinct peaks of prediction scores can be observed solely based on the phastCons score (see Additional file 1: Figure S5A). These scoring peaks can be filtered out by setting a threshold on the minimum number of sequences present in the multi-species alignment (see Additional file 1: Figure S5B-D). This can be explained by high DNA conservation in species not included in our study (such as zebrafish) or just high DNA conservation not preserved on the AA level. Another reason could be the incompleteness of the multi-species alignment itself. Furthermore false negatives due to highly divergent or quickly diverging sORFs cannot be ruled out. Addressing these limitations extends well beyond the scope of this paper. For the time being it merely limits the power of the *in silico* prediction strategy leading to an underestimation of putatively coding sORFs.

Ribosome profiling, an experimental approach to monitor *in vivo* translation by estimating the rate of protein synthesis from the density of ribosome footprints cannot be proposed as a fool-proof method to distinguish between coding and non-coding transcripts. For example, the ncRNA *H19* shows ribosomal occupancy and hence also ribosome profiles, but is nonetheless a non-coding sequence [41]. In addition, one has to keep in mind that spurious association of ribosomes could lead to translational noise and as such most of the transcripts suggested to encode small peptides seem to lack conservation of their proposed coding regions [25]. Recently, Guttmann *et al.* proposed a new metric to distinguish between protein-coding and all classes of non-coding transcripts showing ribosome occupancy [42]. On the other hand, the ribosome profiling technique greatly outperforms mass spectrometry, the commonly applied technique for protein product identification, with regard to dynamic range and comprehensiveness. Presumably, coding sORFs are translated at low levels [5], thus making these properties very important.

**Figure 4:**
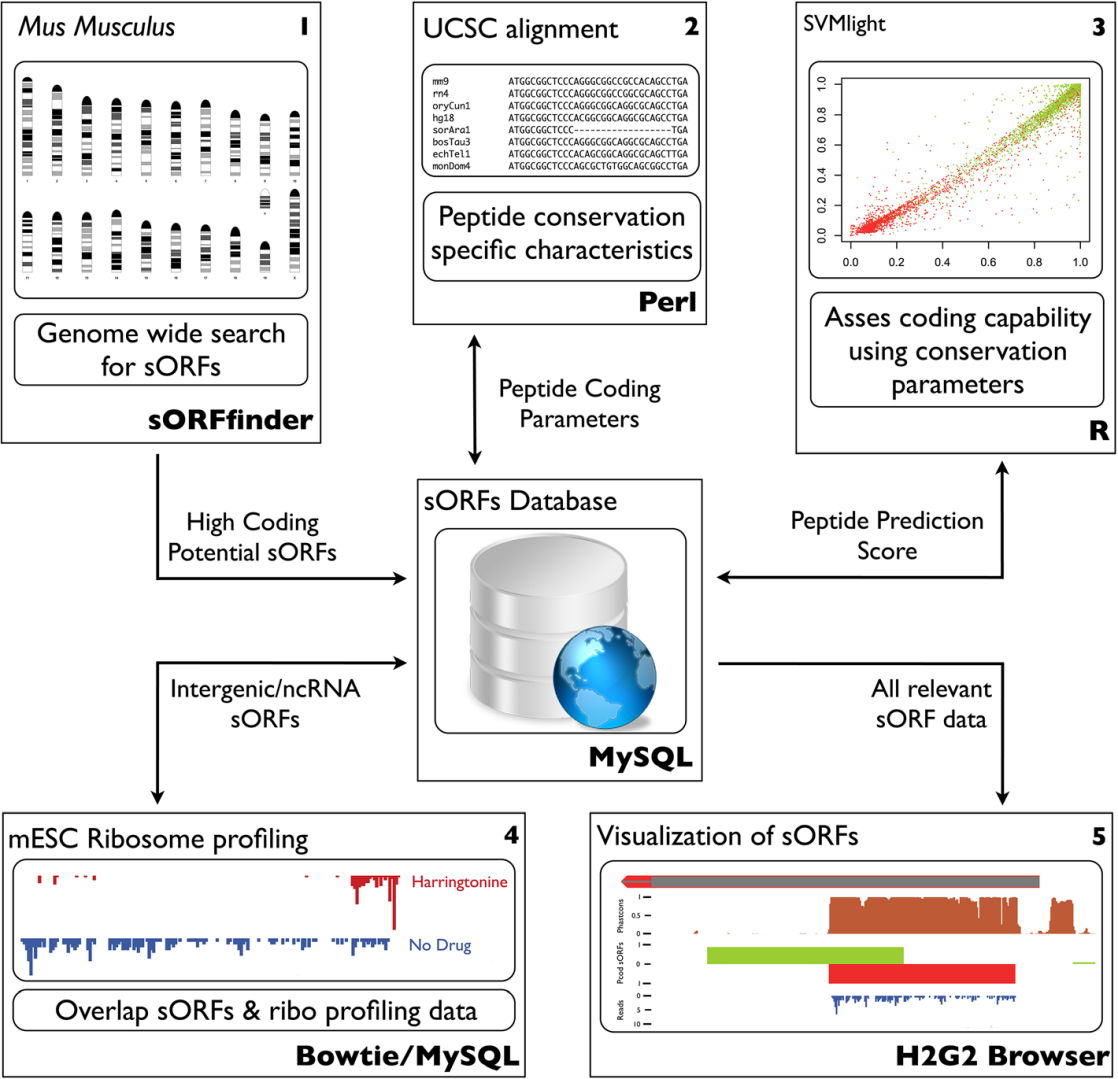
**General layout of the identification pipeline.** The identification pipeline consists of different steps as outlined in the workflow. Central in the analysis is the MySQL sORF database where all obtained and calculated data is stored. This overall sORF data matrix can be downloaded via Additional file 1. **(1)** Genome-wide search for sORFs (with high coding potential) with the sORFfinder package. **(2)** Calculation of different peptide conservation measures based on the UCSC Mouse multiple alignments. **(3)** Coding capability assessment of the sORFs by means of a Support Vector Machine (SVM) learning algorithm. **(4)** Inspection of the sORF locations for presence of ribosome profiling signals obtained from mESC experiments. **(5)** Genome-wide visualization of all (experimental) data and all sORF information on our in-house developed H2G2 Genome Browser.

Our combinatorial pipeline, as outlined in the workflow (see Figure 4), overcomes most of these aforementioned shortcomings. We do not merely identify *in silico* predicted sORFs with high conservation, prone to false positives, or sORFs with translational evidence, for which it is sometimes hard to differentiate between true coding and non-coding. The identification is based on a combination of both measures. Hence, the putative micropeptide-encoding sORFs identified in this report are very good candidates for further *in vitro* and *in vivo* research as they show high conservation at both DNA and (more importantly) AA level in different mammalian species, as well as translation measured by ribosomal occupancy.

Our analysis is certainly no endpoint. As already mentioned, known micropeptides have a very narrow expression window [2,5]. Also, the fact that we only used one cell type (feeder-free cultured E14 mESC) in the overlap with our *in silico* prediction, means that presumably only a small segment of putatively coding sORFs has been identified. This tissue and developmental stage specific expression (that recently was shown to be very notable within the ENCODE project [43]) leaves a lot of sORFs yet to be identified. The more additional ribosome profiling data will be available in the future, the more sORFs will be picked up as interesting starting points for further analysis, after overlap with our prediction information. Next to the advent of more experimental data, the *in silico* analysis can also further improve in the future. Taking into account the computational load to identify all sORFs with an initiator methionine in the *Mus musculus* genome, it would be almost impossible to perform a genome-wide *in silico* scan for all near-cognate sORFs. Following another workflow, whereby we first search for all sORFs showing translation evidence in ribosome profiling studies, and afterwards perform the *in silico* analysis on these candidates could be an appropriate alternative.

## Conclusions

Our combined genome-wide approach towards the identification of sORFs in *Mus musculus*, leads to the prediction of a comprehensive but manageable set of putatively coding sORFs. In this respect, our study is a very important first step towards the identification of a new class of bioactive peptides, called micropeptides. Starting from the described results, further *in vivo* experiments (mass spectromic validation and genetic experiments) should be carried out, testing activity and functionality of the identified peptide products.

## Methods

### General layout of the identification pipeline

The presented pipeline consists of different steps (see Figure 4). First, the genomic sequence was scanned for sORFs (with high coding potential) using the sORFfinder package [32]. Secondly, the conservation pattern of those detected sORFs was investigated by means of the UCSC multiple alignment containing 29 vertebrate genomes for *Mus musculus*[44]. For all sORFs several measures pointing to peptide sequence conservation were gathered from this multiple alignment, leading to a comprehensive data matrix that formed the basis for further mathematical analysis. In a third step, a machine learning technique (more specifically a SVM) was applied assessing the coding capabilities of the sORFs [45]. As such, we were able to classify and subsequently rank the sORFs based on a set of relevant peptide sequence conservation measures. Afterwards, these sORF locations were verified for the presence of ribosome profiling signals, obtained from experiments performed on mESCs. As micropeptides seem to play their role during embryogenesis and morphogenesis, these specific mESC data resources are assumed to be extremely valuable. The overlap of both the *in silico* prediction analysis (resulting in sORFs with high coding potential) and the ribosome profiling data (suggesting translation) could potentially yield functional micropeptides. An in-house developed genome browser (H2G2) was subsequently used to visually inspect all aforementioned (experimental) data on a genome-wide scale (http://h2g2.ugent.be/biobix.html) (see Additional file 1 for login credentials).

### Genome-wide identification of sORFs

sORFfinder was used to search for sORFs in the *Mus musculus* (NCBIM37.66) genome [32], checking for the presence of potentially coding sORFs with a length between 10 and 100 AA. Prior to genome-wide scanning, the hidden Markov model (HMM) was trained with exon (coding) and intron (non-coding) data from the longest chromosome 1. The value for P, reflecting the coding percentage in the mouse genome, was set to 0.025 [46]. The in-house developed script (and all further computational scripting) was run on a 16 core 128 Gb Ram Linux server, running CentOS 5.2. sORFfinder took between 5–14 days analyzing one chromosome, depending on its size, using up to 50 Gb of memory. All sORFs and further obtained data were stored in an InnoDB MySQL (v 5.5.18) database, making use of table partitioning (both List and Hash) and indexing for efficient querying. The sORF data matrix is also available as a downloadable CSV file via Additional file 1.

### Peptide conservation based on UCSC multiple species alignment

The multiple species alignment used in this analysis was obtained from UCSC [44]. The *Mus musculus* mm9 multiple species alignment contains 29 species of which 8 (relevant for this study) were chosen: *Rattus norvegicus, Cavia porcellus, Oryctolagus cuniculus, Homo sapiens, Sorex araneus, Bos taurus, Echinops telfairi,* and *Monodelphis domestica.* Custom scripts (Perl v.5.8.8) were applied to extract the alignment block for each sORF in order to distill relevant peptide conservation characteristics.These characteristics include the number of aligned sequences, the number of alignments having a conserved ORF length, the number of alignments with conserved start and stop codon, and the total amount of synonymous versus non-synonymous mutations between the different species as compared to *Mus musculus*. Using the BioPerl package Bio: Align: DNAStatistics (available from CPAN, http://search.cpan.org/~cjfields/BioPerl-1.6.901/Bio/Align/DNAStatistics.pm), pair-wise K_a_/K_s_ values were calculated using the function “calc_kaks_pair” based upon the Nei-Gojobori statistics [47].

### SVM trainings data, classification and ranking

SVM^light^ is an implementation of SVMs in C. SVM^light^[45] was used within the R-project (R v 2.12.2, [48]) package klaR (v 0.6-6, [49]) available via CRAN (http://cran.r-project.org/web/packages/mixOmics/index.html). LibSVM [50] is made available both as a C++ and Java software package for support vector classification which can be used through the R package kernlab (v 0.9-14) [51] and is also available via CRAN (http://cran.r-project.org/web/packages/kernlab/index.html). An R-script was compiled to train the SVMs and subsequently classify and rank all the obtained sORFs according to coding probability. Both SVMs were run with a linear kernel and standard parameters.

The negative, non-coding training data were constructed from predicted sORFs, located in annotated intronic regions of known protein-coding genes. A set of randomly constructed DNA-sequences was used as positive coding training data, having the same length distribution as the predicted sORFs. The sequences are located within annotated exonic regions of known genes. The positive training data had to be in frame with the protein-coding part of a gene to mimic true conservation at the AA level. Therefore, the predicted sORFs located in known exonic regions could not be used, as the greater majority of the sORFs were not in frame with the protein coding part of the gene in which they are located. The training data (48,196 sequences) consisted of an equal amount of coding and non-coding sequences, randomly selected from all available training data. SVMs were trained on 4/5^th^ of the data, and tested on the remaining sequences. Further cross-validation did not improve nor change overall classification and ranking.

### mESC ribosome profiling data

Raw sequencing data of the mESC ribosome profiling data [16] were downloaded from the Gene Expression Omnibus (dataset GSE30839). All reads from the normal (cycloheximide-treated, sample GSM765292) and harringtonine-treated (sample GSM765295) were remapped using bowtie (v. 0.12.7) on the mouse genome (assembly version 37). After removal of rRNA mapped reads, the remaining reads were used to analyze intergenic and ncRNA sORFs. For evaluation of the intergenic sORFs, reads were first mapped on the mouse cDNA database (Ensembl version 66). Unmapped reads were subsequently mapped on a custom database constructed from all intergenic sORF sequences. At both the 5′ and 3′ ends these intergenic sORF sequences were extended with 20 bases, thus also allowing ribosome profile mapping at both termini (an offset is generally applied for ribosome profile mapping [16]). For ncRNA sORF evaluation, reads were uniquely mapped to the mouse genome after removal of rRNA mapped reads. All sORFs from both aforementioned sets were first investigated for the presence of ribosome profile peaks at the translation start position, based upon the harringtonine-treated data. Secondly, the sequencing data from the cycloheximide-treated sample were used to calculate overall coverage (based on total read length) and an RPKM value. The RPKM value is defined as the total number of reads mapped to the sORF per kb sORF exon sequence divided by the total amount of non-rRNA reads (in million reads). Only sORFs with a coverage > 75% where retained for further analysis. The 75% threshold is based on the mean value of the fraction-non-zero measures calculated by Ingolia *et al.* 2011 for all the 90 bp windows showing ribosome profile coverage within lincRNAs.

### Genome-wide visualization

Genome-wide visualization of publicly available and experimental data was accomplished by using an in-house developed genome browser (H2G2_,_http://h2g2.ugent.be/biobix.html). Several information tracks are available, including genomic information from a local Ensembl instance (NCBIM37.66), sORF prediction results, phastCons conservation scores, and different experimental results [16,25,35]. The underlying data are stored in a MySQL database (v 5.0.27) enabling genome-wide specific querying and filtering through standard query language (SQL) statements.

## Competing interests

The authors declare that they have no competing interests.

## Authors’ contributions

JC and GM designed the experiments, obtained data, analyzed and interpreted data and wrote the manuscript. GT helped with the visualization of the data. WVC, GB, WL and EH helped with the conception and design of the experiments and critically reviewed the manuscript. All authors read and approved the final manuscript.

## Supplementary Material

Additional file 1 **Supplemental Information.** Contains login credentials to access the H2G2 Genome Browser, data access to the complete sORFs database (690 mb) and Figures S1 to S5. Click here for file

Additional file 2 **ncRNA and intergenic sORFs with ribosome profiling evidence.** Contains all sORFs overlapping ncRNA or intergenic regions for which ribosomal profiling evidence exists. Included are the genomic locations, all peptide conservation characteristics and coding potential score. Click here for file
