# Supplementary material for: Combining in silico prediction and ribosome profiling in a genome-wide search for novel putatively coding sORFs
Source: BMC Genomics. 2013 Sep 23;14:648. doi: 10.1186/1471-2164-14-648 (PMC3852105; doi:10.1186/1471-2164-14-648)
Supplement: Additional file 1 — Supplemental Information. Contains login credentials to access the H2G2 Genome Browser, data access to the complete sORFs database (690 mb) and Figures S1 to S5. [file 1471-2164-14-648-S1.pdf]

Supplemental Information

H2G2 login credentials

The H2G2 Genome Browser can be accessed via the URL http://h2g2.ugent.be/biobix.html using the following login credentials:

Name: micropeptides
Password: m!cr0pept!des

In the upper left corner under "Select Project" one can choose "Mouse micropeptides". The genome browser software lets you zoom from the chromosome to the nucleotide level in a semantic way, as such data is loaded and displayed according to the zooming level and region displayed.

Extra information in the form of static and sample tracks can be dragged into the visualization field from the left.

Further information on the usage of the H2G2 genome browser can be found in the Manual which can be downloaded here: http://h2g2.ugent.be/downloads/ManualV1_0.pdf.

sORFs Database

The complete sORFs database contains all the identified *Mus musculus* sORFs with all deduced peptide conservation characteristics and coding potential score. This database can be downloaded via the URL http://h2g2.ugent.be/share/sorfsdatabase/ using the following login credentials:

Name: micropeptides
Password: m!cr0pept!des

```
TRANSCRIPT

        Chr:      2
        Begin:    127618033
        End:      127618203
        Strand:   -1


GENERAL OVERVIEW

        Alignment found:              7/8
        Conserved length:             7/7
        Conserved start and stop:     7/7


MUTATIONS OVERVIEW S=Synonymous, N=Non-Synonymous, P=Premature Stopcodon

        Species       S     N     P

        mm9           0     0     0
        rn4           5     0     0
        cavPor2       16    3     0
        oryCun1       11    5     0
        hg18          17    3     0
        bosTau3       20    5     0
        echTel1       15    7     0
        monDom4       16    27    0


AA ALIGNMENT WITH CONSERVATION LINE

        mm9           MADVSERTLQVSVLVAFASGVVLGWQANRLRRRYLDWRKRRLQDKLATTQKKLDLA*
                               S            S      S    S           S
        rn4           MADVSERTLQVSVLVAFASGVVLGWQANRLRRRYLDWRKRRLQDKLATTQKKLDLA*
                        S S      S SNS  S  NN    S S  S S   SSS     S S S
        cavPor2       MADVSERTLQVSVLLAFASGVLVGWQANRLRRRYLDWRKRRLQDKLATTQKKLDLA*
                          S S  N S S    S  NN  S S    S S           SNN S
        oryCun1       MADVSERTLRVSVLVAFASGVLVGWQANRLRRRYLDWRKRRLQDKLAVMQKKLDLA*
                        S S  S  N   SS S  SN   S SS   S S   SS      SNS S
        hg18          MADVSERTLQLSVLVAFASGVLLGWQANRLRRRYLDWRKRRLQDKLAATQKKLDLA*
                          S  S  NSSS S  S  NN  SSS   SS S    N  SS SSNS S  S
        bosTau3       MADVSERTLQLSVLVAFASGVLVGWQANRLRRRYLDWRKKRLQDKLAATQKKLDLA*
                          S  S  N  SSS  SNNNN    S S  S S    N      SNS S S S
        echTel1       MADVSERTLQFSVLVAFASRILVGWQANRLRRRYLDWRKKRLQDKLAVTQKKLDLA*
                       NNNNNSNNN NSNSN SSSNNN  S NSSNSSNSN S NS  NN NNS NNS NN
        monDom4       MKEIGDRKVRVAVVVSFASGFFVGWQACRLWRRFLNWRKGRLQEQLQETQRRLDMY*


DNA ALIGNMENT

        mm9           ATGGCGGACGTGTCTGAGAGGACGCTGCAGGTGTCCGTGCTAGTGGCTTTCGCCTCTGGAGTGGTCCTGGGCTGGCAAGCGAATCGGCTGCGGAGGCGTTACCTAGACTGGAGGAAGCGGAGGCTGCAGGACAAGCTGGCAACGACTCAGAAAAAGCTGGACCTGGCCTGA
        rn4           ATGGCGGACGTGTCTGAGAGGACGCTGCAAGTGTCCGTGCTAGTGGCTTTCGCCTCTGGAGTGGTCCTTGGCTGGCAAGCGAATCGGTTGCGGAGGCGTTACCTGGACTGGAGGAAGCGGAGGCTGCAGGACAAGCTGGCGACGACTCAGAAAAAGCTGGACCTGGCCTGA
        cavPor2       ATGGCGGATGTGTCGGAGAGGACGCTGCAGGTGTCGGTGCTGCTGGCCTTCGCCTCCGGAGTGCTCGTGGGCTGGCAAGCGAACCGGTTGCGGAGGCGCTACCTGGACTGGAGGAAACGAAGACTGCAGGACAAGCTGGCCACGACGCAGAAGAAGCTGGACCTGGCCTGA
        oryCun1       ATGGCGGACGTGTCCGAGCGCACGCTGCGAGTGTCGGTGCTGGTGGCTTTCGCCTCCGGAGTGCTCGTGGGCTGGCAGGCGAACCGGCTGCGGAGGCGCTACCTGGACTGGAGGAAGCGGAGGCTGCAGGACAAGCTGGCGGTGATGCAGAAGAAGCTGGACCTGGCCTGA
        hg18          ATGGCGGATGTGTCAGAGAGGACACTGCAGTTGTCCGTGCTAGTAGCCTTCGCTTCTGGAGTACTCCTGGGCTGGCAGGCGAACCGACTGCGGAGGCGCTACTTGGACTGGAGGAAAAGGAGGCTGCAGGACAAGCTGGCGGCGACGCAGAAGAAGCTGGACCTGGCCTGA
        bosTau3       ATGGCGGACGTGTCCGAGAGGACACTGCAGTTGTCTGTACTGGTGGCATTCGCCTCCGGAGTGCTCGTGGGCTGGCAGGCAAACCGGCTGCGGCGGCGCTACCTGGACTGGAGGAAGAAGAGGCTGCAAGATAAGCTAGCGGCGACGCAGAAGAAGCTGGATCTGGCCTGA
        echTel1       ATGGCGGACGTGTCGGAGAGGACCCTGCAGTTCTCCGTGCTTGTAGCGTTCGCCTCCCGAATCCTCGTGGGCTGGCAAGCGAACCGGCTCCGGAGGCGCTACTTGGACTGGAGGAAGAAGAGGCTGCAGGACAAGCTGGCGGTGACACAGAAGAAGTTGGACCTAGCCTGA
        monDom4       ATGAAGGAGATAGGAGATCGGAAGGTGCGCGTGGCCGTAGTGGTCTCCTTCGCTTCGGGCTTCTTCGTAGGCTGGCAGGCGTGCCGCTTATGGAGACGCTTCCTGAACTGGCGGAAGGGGCGGCTGCAGGAACAGCTGCAAGAGACGCAGAGGCGGTTGGACATGTACTGA
```

Figure S1 Peptide conservation parameters based on UCSC multi-species alignment

Example of an overview file generated for each sORF. This file contains the DNA and AA multiple alignments for the sORF and serves as a starting point for the calculation of all peptide conservation characteristics, which can be found in the file itself but also in the sORF database (See Supplemental Data File 2). Species in the multiple alignment: *Mus Musculus* (mm9), *Rattus norvegicus* (rn4), *Cavia porcellus* (cavPor2), *Oryctolagus cuniculus* (oryCun1), *Homo sapiens* (hg18), *Bos taurus* (bosTau3), *Echinops telfairi* (echTel1), and *Monodelphis domestica* (monDom4)

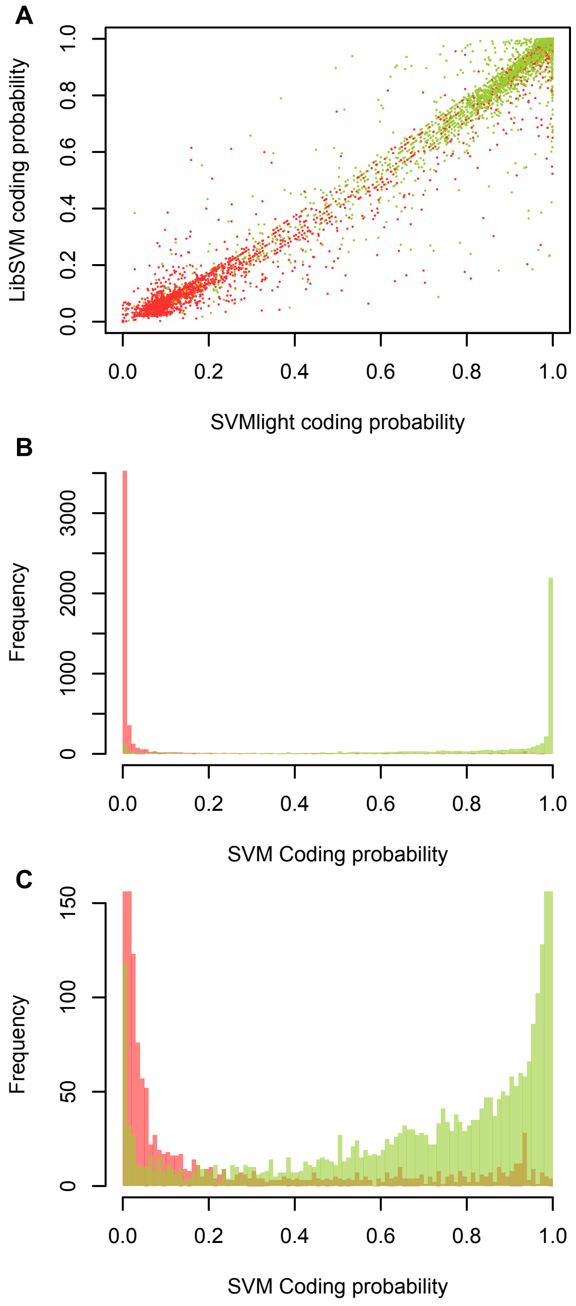

Figure S2 Classification of test data using SVMs

(A) Visual representation of the classification of all 9,612 test subjects, based upon both SVMs (SVM^{light} and LibSVM, see also material and methods section). True coding subjects are depicted in green and true non-coding in red. (B) and (C) Histograms of SVM coding probability based upon the SVM^{light} analysis. All red sORFs with coding probability above 0.5 are in fact false positives; all green sORFs having a score lower as 0.5 are false negatives. See also figure 1C for visual interpretation of all false/true negative/positive test subjects.

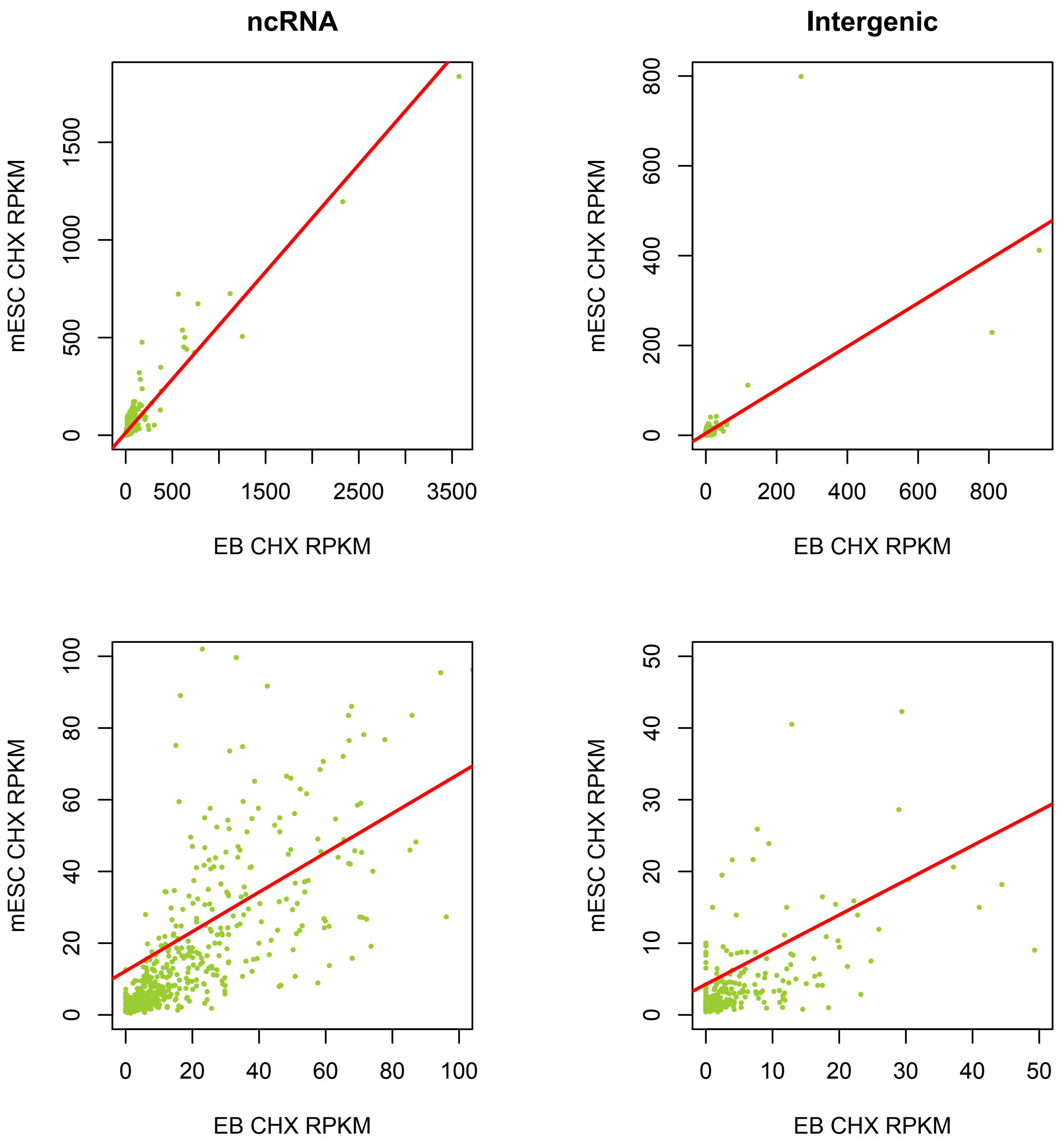

Figure S3 Comparison of expression between mESC CHX and EB CHX treated samples

Representation of all ncRNA and intergenic sORFs with ribosomal mESC CHX read coverage > 0.75 and harringtonine treated mESC ribosome profiling occupancy. A scatterplot comparing the mESC CHX RPKM value and the corresponding EB RPKM value is shown for both sets of sORFs. The red line is a linear regression trend line based on the RPKM of the mESC CHX versus the RPKM of the EB CHX sample data. The exact coverage and RPKM values for each of the ncRNA and intergenic sORFs with ribosome footprint occupancy can be found in additional file 2.

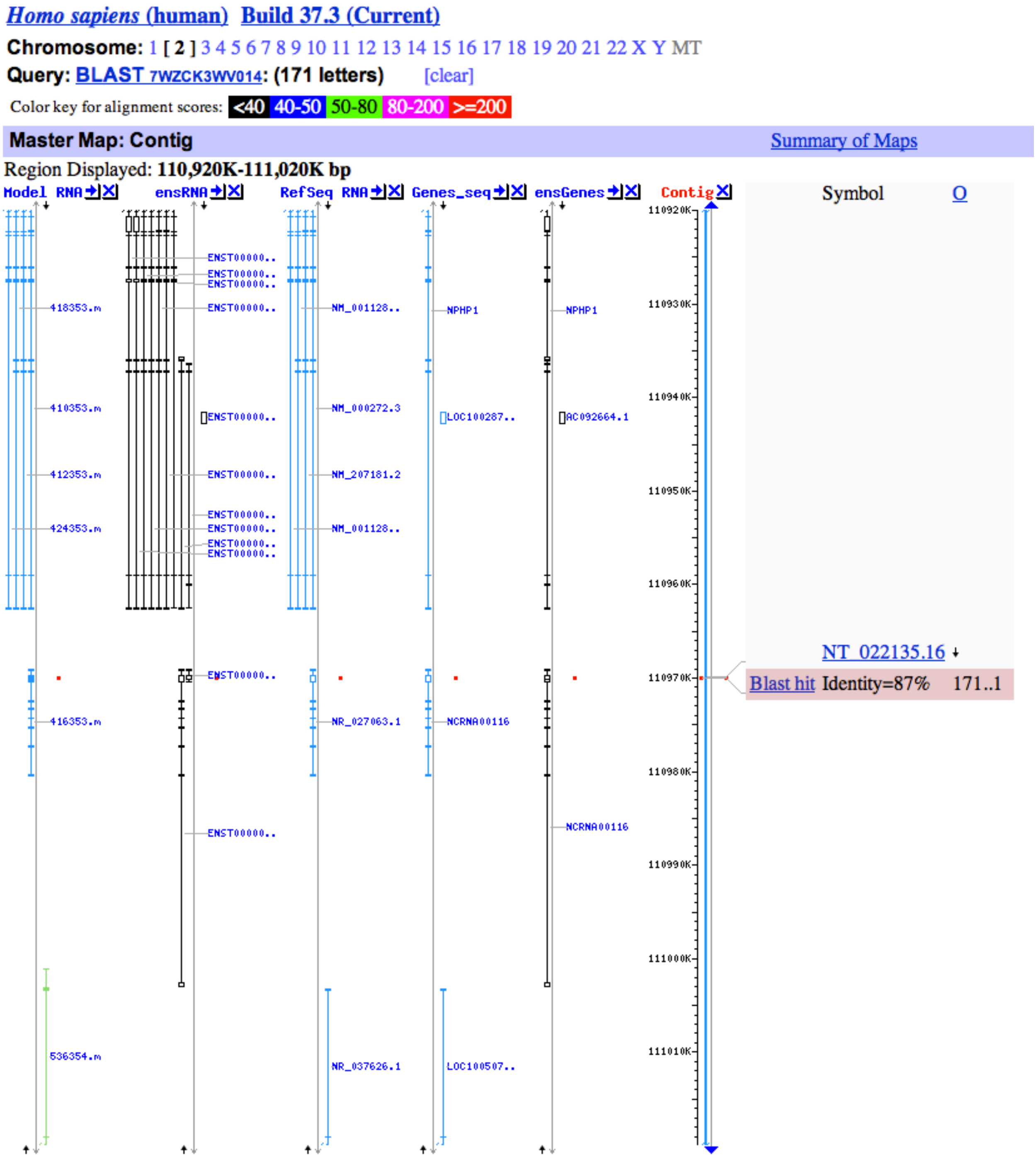

Figure S4 Blast result for a lincRNA overlapping sORF against the human genome

A discontiguous megablast (blastn) against the human genome for the sORF overlapping a lincRNA (1500011K16Rik) as presented in figures 3A, 3C and S1 lead to the identification of 1 region in the human genome. This region is located within the second and biggest exon of the lincRNA *linc00116.*

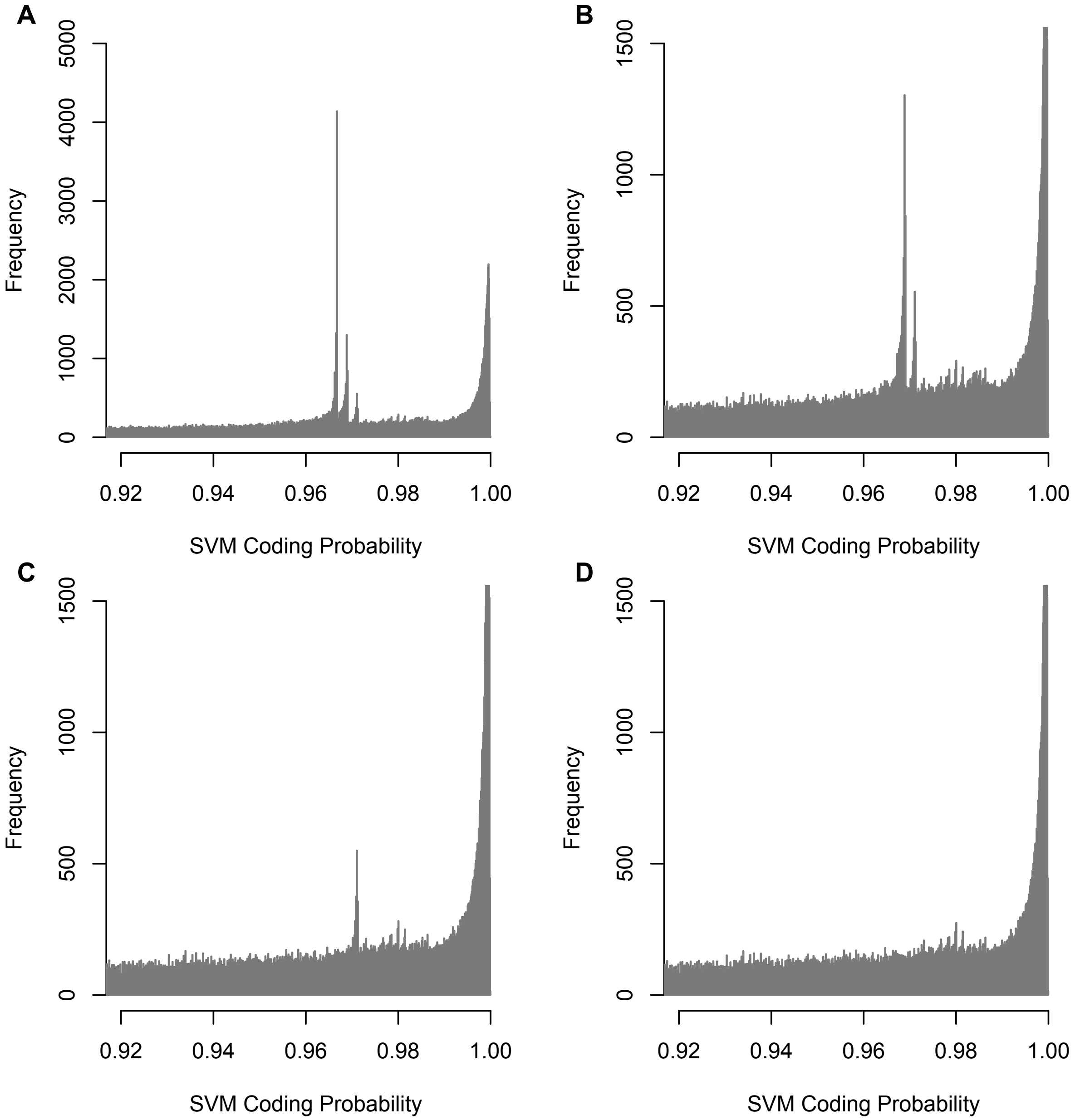

Figure S5 Influence of phastCons score on classification of sORFs

sORFs having no or very few aligned sequences (within the set of 8 pair-wise alignments under investigation), lack most peptide conservation characteristics which are necessary within the *in silico* pipeline to thoroughly assess their coding probability. As a consequence the classification for this group of sORFs is mainly based on the phastCons conservation score, a measure for DNA conservation. This leads to the arbitrary peaks at certain coding probability scores as a lot of those sORFs still have high phastCons scores, mostly based on alignments not under

investigation. When a cut-off is placed on the minimal number of sequence alignments that have to be present for coding probability interpretation, these peaks tend to disappear completely and only those sORFs remain for which DNA as well as AA conservation can be thoroughly assessed.

(A) Histogram of the coding probability for all sORFs as based upon SVM^{light}. (B) Histogram of the coding probability for all sORFs having at least 1 alignment within the 8 species under investigation. (C) Histogram of coding probability for all sORFs having at least 2 alignments. (D) Histogram of coding probability for all sORFs with at least 3 alignments.
